# Bioinspired Rhamnolipid Protects Wheat Against *Zymoseptoria tritici* Through Mainly Direct Antifungal Activity and Without Major Impact on Leaf Physiology

**DOI:** 10.3389/fpls.2022.878272

**Published:** 2022-06-03

**Authors:** Rémi Platel, Anca Lucau-Danila, Raymonde Baltenweck, Alessandra Maia-Grondard, Ludovic Chaveriat, Maryline Magnin-Robert, Béatrice Randoux, Pauline Trapet, Patrice Halama, Patrick Martin, Jean-Louis Hilbert, Monica Höfte, Philippe Hugueney, Philippe Reignault, Ali Siah

**Affiliations:** ^1^Joint Research Unit 1158 BioEcoAgro, Junia, Univ. Lille, Univ. Liège, UPJV, Univ. Artois, ULCO, INRAE, Lille, France; ^2^Université de Strasbourg, INRAE, SVQV UMR A1131, Colmar, France; ^3^Univ. Artois, UniLasalle, ULR 7519–Unité Transformations and Agroressources, Béthune, France; ^4^Unité de Chimie Environnementale et Interactions sur le Vivant (EA 4492), Université du Littoral Côte d'Opale, Calais, France; ^5^Laboratory of Phytopathology, Department of Plants and Crops, Ghent University, Ghent, Belgium

**Keywords:** wheat, *Zymoseptoria tritici*, rhamnolipids, plant defenses, transcriptomic, metabolomic, omics

## Abstract

Rhamnolipids (RLs), glycolipids biosynthesized by the *Pseudomonas* and *Burkholderia* genera, are known to display various activities against a wide range of pathogens. Most previous studies on RLs focused on their direct antimicrobial activity, while only a few reports described the mechanisms by which RLs induce resistance against phytopathogens and the related fitness cost on plant physiology. Here, we combined transcriptomic and metabolomic approaches to unravel the mechanisms underlying RL-induced resistance in wheat against the hemibiotrophic fungus *Zymoseptoria tritici*, a major pathogen of this crop. Investigations were carried out by treating wheat plants with a bioinspired synthetic mono-RL with a 12-carbon fatty acid tail, dodecanoyl α/β-L-rhamnopyranoside (Rh-Est-C12), under both infectious and non-infectious conditions to examine its potential wheat defense-eliciting and priming bioactivities. Whereas, Rh-Est-C12 conferred to wheat a significant protection against *Z. tritici* (41% disease severity reduction), only a slight effect of this RL on wheat leaf gene expression and metabolite accumulation was observed. A subset of 24 differentially expressed genes (DEGs) and 11 differentially accumulated metabolites (DAMs) was scored in elicitation modalities 2, 5, and 15 days post-treatment (dpt), and 25 DEGs and 17 DAMs were recorded in priming modalities 5 and 15 dpt. Most changes were down-regulations, and only a few DEGs and DAMs associated with resistance to pathogens were identified. Nevertheless, a transient early regulation in gene expression was highlighted at 2 dpt (e.g., genes involved in signaling, transcription, translation, cell-wall structure, and function), suggesting a perception of the RL by the plant upon treatment. Further *in vitro* and *in planta* bioassays showed that Rh-Est-C12 displays a significant direct antimicrobial activity toward *Z. tritici*. Taken together, our results suggest that Rh-Est-C12 confers protection to wheat against *Z. tritici* through direct antifungal activity and, to a lesser extent, by induction of plant defenses without causing major alterations in plant metabolism. This study provides new insights into the modes of action of RLs on the wheat-*Z. tritici* pathosystem and highlights the potential interest in Rh-Est-C12, a low-fitness cost molecule, to control this pathogen.

## Introduction

Biosurfactants, also referred to as green surfactants, are amphiphilic surface-active molecules that contain a hydrophilic head and a hydrophobic tail (Desai and Banat, 1997). They are produced by a wide range of microorganisms, including bacteria, yeasts, and fungi, and are classified according to their molecular structure into mainly mannosylerythritol lipids, trehalose dimycolate, trehalolipids, sophorolipids, lipopeptides, and rhamnolipids (Crouzet et al., 2020). Biosurfactants have been extensively studied for decades because of their strong potential for application in various fields, including human health, cosmetics, food industry, petroleum industry, soil and water remediation, nanotechnology, and agriculture (Lourith and Kanlayavattanakul, 2009; Sachdev and Cameotra, 2013; Shekhar et al., 2015; Singh et al., 2019). Besides, these biomolecules are gaining more and more attention because of their high biodegradability as well as low toxicity and ecotoxicity, making them an eco-friendly alternative to synthetic molecules in several areas, in particular for sustainable agriculture (Abdel-Mawgoud et al., 2010; Shekhar et al., 2015).

Rhamnolipids (RLs) are glycolipids with biosurfactant properties, naturally biosynthesized as secondary metabolites by bacteria from the *Pseudomonas* and *Burkholderia* genera. The first report on RLs goes back to 1946, from an *in vitro* culture of *Pseudomonas pyocyanea* (now *Pseudomonas aeruginosa*) (Bergström et al., 1946). Since then, RLs have been among the most intensively studied classes of surfactant glycolipids. Bacteria often produce RLs as a mixture of molecules with one or two rhamnose residues (mono- or di-RLs), forming a polar hydrophilic head and linked through a beta-glycosylic bond to one or two 3-hydroxy fatty acids as hydrophobic tails. Because of high natural variation in their chemical structures, analyses of bacterial RLs have led to the identification of a pool of almost 60 different RLs (Abdel-Mawgoud et al., 2010). Differences among these homologs come from modifications in glycon and/or aglycon parts; however, the biodiversity of RLs is mostly due to variations in aglycon parts (Abdel-Mawgoud et al., 2010). The potential efficacy of RLs in biocontrol of plant pathogens has been reported in several studies, as recently reviewed by Crouzet et al. (2020). Most studies describe the activities of RLs against a large panel of phytopathogenic fungi and oomycetes, but no direct evidence has been reported on their bioactivities toward bacteria and viruses infecting plants (Crouzet et al., 2020). The mode of action of RLs may be direct toward pathogens, through antifungal activity, and/or indirect by induction of plant immunity reactions, as was, for instance, recently reported on the *Brassica napus*-*Leptosphaeria maculans* pathosystem using semi-purified RL mixtures from *P. aeruginosa* (Monnier et al., 2020).

Compounds activating plant defense reactions against biotic stress can be grouped into two categories, elicitors or priming compounds, and both can be referred to as resistance inducers. Elicitors induce plant defense responses immediately after their perception by a plant, whereas priming agents usually activate marked defense responses, not directly after their application but only once the plant is attacked by a pathogen, making priming agents more suitable than elicitors because of their expected lower fitness cost on plant physiology (Paré et al., 2005). Primed plants set up stronger and/or faster response patterns and may detect a pathogen attack at a lower threshold, hence reacting in a more sensitized way when compared to naïve (unprimed) plants (Paré et al., 2005; Lämke and Bäurle, 2017). Activation of plant defense responses could rely on either specific recognition by cell surface immune, of conserved molecular motifs from a pathogen, the plant itself or a beneficial microbe (Jones and Dangl, 2006), or on exogenous application of natural or synthetic molecules, possibly not perceived by specific plant transmembrane receptors, such as phytohormones and biosurfactants (Siah et al., 2018). Perception of initial stimuli by a plant often leads to the onset of numerous defense mechanisms, including earlier (influx of calcium, rapid extracellular alkalinization, mitogen-activated protein kinase cascade involved in phosphorylation, oxidative burst, etc.) and subsequent cellular events (transcriptional changes in attack-responsive genes, biosynthesis of pathogenesis-related (PR) proteins, production of low-molecular mass secondary metabolites, such as phytoalexins or antimicrobial peptides, etc.) (Bolwell et al., 1995; Felle, 2001; Pedley and Martin, 2005; van Loon et al., 2006; Galon et al., 2010).

Wheat, along with maize and rice, is one of the most cultivated crops worldwide. It is used as a primary ingredient for both food and feed as well as in several agro-industrial areas. Nevertheless, this crop is challenged in the field by several bio-aggressors, which significantly impact wheat grain quality and quantity (Savary et al., 2019). Septoria tritici blotch, caused by the fungal ascomycete *Zymoseptoria tritici*, is, for few decades, one of the most frequently occurring and damaging diseases on the wheat crop, causing yield losses of up to 50% especially in areas where environmental conditions are suitable for disease establishment (Fones and Gurr, 2015). *Z. tritici* is a hemibiotrophic fungus characterized by an initial asymptomatic biotrophic phase lasting approximately 2 weeks, followed by a visually symptomatic necrotrophic phase of around one week (Siah et al., 2010). However, the duration of these two phases may vary depending on wheat cultivar, fungal strain, and environmental conditions. The control of *Z. tritici* relies mainly on the use of chemical fungicides, such as in Europe, where 70% of total applied fungicides target this pathogen (Fones and Gurr, 2015) and, to a lesser extent, resistant cultivars, since no cultivar completely resistant to *Z. tritici* is available. Meanwhile, the durability of these management strategies is often compromised in the field, since *Z. tritici* frequently develops resistance to fungicides and regularly overcomes host resistance (e.g., Cowger et al., 2000; Cheval et al., 2017). Hence, looking for new eco-friendly and sustainable protection alternative tools for wheat protection against *Z. tritici*, such as bio-sourced resistance inducers, is strongly encouraged. However, the literature on induced resistance in wheat against this major fungal pathogen is still poor, while defense mechanisms of wheat that are triggered in response to *Z. tritici* infection are extensively studied at the plant-pathogen interaction level (e.g., Rudd et al., 2015; Seybold et al., 2020). The objective of this study was, thus, to unravel defense mechanisms induced in wheat toward *Z. tritici* by a bioinspired synthetic mono-RL with a C12-carbon chain (Rh-Est-C12), which was recently shown to be the most active and protective RL among a series of 19 RLs tested on this pathosystem by a structure-activity relationship study (Platel et al., 2021). The investigations were performed under greenhouse conditions using a combined transcriptomics and metabolomics approach under both noninfectious and infectious conditions to assess the potential eliciting and priming effects of the molecule. Interestingly, the results showed that RLRh-Est-C12 confers protection to wheat against *Z. tritici* without inducing major changes in wheat leaf physiology.

## Materials and Methods

### Bioinspired Rhamnolipid Synthesis

**Figure 1:**
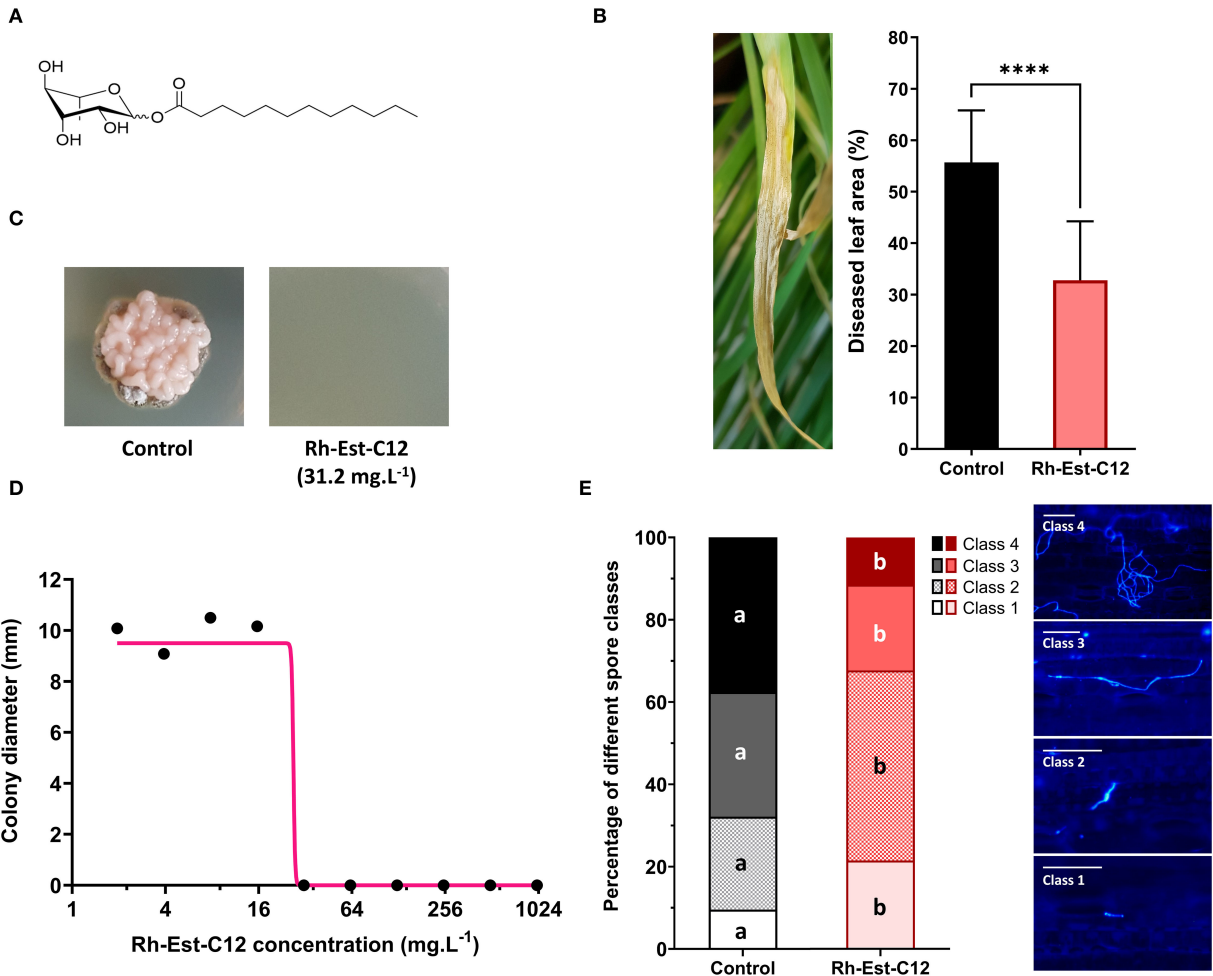
Protective and antifungal effects of Rh-Est-C12 on wheat (Cv. Alixan) toward *Zymoseptoria tritici* (T02596 strain). **(A)** Molecular structure of the bioinspired synthetic rhamnolipid (RL) Rh-Est-C12 used in this study. The RL is composed of rhamnose linked to a C12-carbon chain with an ester bond. **(B)** Disease severity level of Septoria tritici blotch assessed 23 days post-treatment (D23) with Rh-Est-C12 at 500 mg.L^−1^ or a mock solution, *i.e*., 21 days post-inoculation with the pathogen, by scoring the area of chlorosis and necrosis on wheat third leaves. Bars stand for standard-deviation (*n* = 36). Data were analyzed by unpaired *t*-test (*P* ≤ 0.05). A representative picture of disease symptoms on D23 in non-treated wheat is shown on the left of the Figure. **(C)** Pictures illustrating colonies in the following *in vitro* assay. **(D)** *In vitro* antifungal dose-response curve of Rh-Est-C12 against *Z. tritici* (T02596 strain). The direct antifungal activity of the rhamnolipid against the fungal pathogen was assessed by measuring perpendicular diameters of *Z. tritici* colonies after 10 days on PDA medium plates supplemented with different concentrations of the RL (*n* = 6). Non-linear regression was designed using GraphPad Prism software version 9. **(E)** *In planta* epiphytic spore development state (class 1: non-germinated spore; class 2: germinated spore with small germ tube; class 3: germinated spore with developed germ tube; class 4: germinated spore with a strongly developed germ tube) was assessed using Calcofluor dye 5 days after treatment with Rh-Est-C12 at 500 mg.L^−1^, *i.e*., 3 days after inoculation. The class of 100 distinct spores, selected randomly, was determined, for each condition (*n* = 9). In each spore class, the presence of different letters indicates a significant difference according to Tukey's test (*P* ≤ 0.05). Scale bar = 25 μm. The **** symbol indicates the significant statistical difference with *P* value < 0.0001.

The RL used in this study (dodecanoyl α/β-L-rhamnopyranoside, hereafter referred to as Rh-Est-C12) is bioinspired from RLs naturally produced by *P. aeruginosa* (Figure 1A) and was synthesized as described previously by Robineau et al. (2020) and Platel et al. (2021). The conversion and purity of the product were assessed by nuclear magnetic resonance (NMR) spectroscopy, and confirmatory analyses were performed by elemental analysis (vario MICRO cube CHNS/O; Elementar, Lyon, France) and NMR spectroscopy (NMR, Bruker 400 MHz spectrometer). Briefly, chemical analysis of the obtained molecule Rh-Est-C12 consists of: yellowish oil (β/α ratio 3:7); Rf 0.48 (AcOEt/MeOH 9/1); ^1^H NMR (CDCl_3_): 5.09–4.77 (m, 2H), 4.06–3.87 (m, 4H), 3.66–3.59 (m, 2H), 3.48–3.41 (m, 2H), 2.4–2.33 (m, 4H), 1.65–1.58 (m, 4H), 1.3–1.25 (m, 38H),0.92 (m, 6H, CH_3_) ^13^C NMR (CDCl_3_): δ 174.7, 172, 93.5, 92.8, 75.9, 74.2, 73.4, 72.6, 71.3, 69.9, 68.7, 68.1, 34.4, 34,3, 32–21.6 (CH_2_ alkyl chain), 17.6, 17.4, 13.6. The molecular weight of the obtained molecule was 346 g.mol^−1^.

### Plant and Fungal Materials

Experiments were conducted using seeds of the wheat cultivar (cv.) Alixan, which is susceptible to *Z. tritici* and provided by the breeding company Limagrain Europe (Verneuil l'Etang, France). All *in vitro* and *in planta* bioassays were carried out using the pathogenic *Z. tritici* single-spore strain T02596, isolated in 2014 in Northern France from a wheat field not treated with fungicides. Since its isolation, the strain was stored in cryotube aliquots at −80°C to avoid genetic drift of the strain due to *in vitro* subcultures. The fungal inoculum was produced by growing the strain in a potato dextrose agar (PDA) medium for 1 week under dark conditions at 20°C.

### *In vitro* Antifungal Bioactivity Assay

The direct activity of Rh-Est-C12 toward *Z. tritici* growth was assessed in the PDA medium, as described by Platel et al. (2021), at different concentrations (1.9, 3.9, 7.8, 15.6, 31.2, 62.5, 125, 250, 500, and 1,000 mg.L^−1^) of the RL. Briefly, the RL was first dissolved in DSMO (0.1 % final DMSO concentration in the medium) before being mixed at approximately 40°C with an autoclaved PDA medium. The experiment was carried out using sterile 12-well plates (Cellstar standard^®^ Greiner Bio-One GmbH, Kremsmünster, Austria); each well was filled with 3 ml of the mixture, and each treatment was replicated three times. Plate inoculation was performed by spotting a drop of 5 μl of *Z. tritici* spore suspension at 5.10^5^ spores.ml^−1^ at the center of each well. Control wells, filled or not with 0.1 % DMSO and inoculated or not with fungal spores, were used. After 10-day incubation at 20 ±°C under dark conditions, fungal growth was evaluated by measuring the two perpendicular diameters of each fungal colony.

### Plant Treatment and Infection

Wheat seeds were first pregerminated in square Petri dishes (12 × 12 cm) on moist filter paper, as described by Siah et al. (2010), before being transferred into 3-L pots filled with universal loam (Gamm Vert, France) and placed in a greenhouse at 21 ± 2°C with a 16-/8-h day-night cycle. Three pots of 12 wheat plants each (*n* = 36) were used as replicates for each modality. After 3 weeks of plant growth, i.e., when third leaves were fully expanded (corresponding to day 0, D0), plants were pretreated with Rh-Est-C12 at 500 mg.L^−1^ or with a mock treatment as control using a hand sprayer. A volume of 30 ml was used to treat each pot. Rh-Est-C12 was first dissolved in dimethyl sulfoxide (DMSO: Sigma-Aldrich, Saint Louis, United States), with a final concentration of DMSO of 0.1% in the treatment solution, supplemented with 0.05% of polyoxyethylene-sorbitan monolaurate (Tween 20; Sigma-Aldrich, Saint Louis, United States) as a wetting agent. The mock treatment consisted of a solution of 0.1% DMSO supplemented with 0.05% of Tween 20. Two days post-treatment (D2), both RL-treated and mock-treated plants were inoculated by spraying 30 ml of 10^6^ spores.mL^−1^*Z. tritici* suspension supplemented with a 0.05 % Tween 20 solution. Non-inoculated plants were mock-inoculated by spraying 30 ml of a 0.05 % Tween 20 solution on the plants of each pot. After inoculation, the plants were covered with a clear polyethylene bag for 3 days to ensure high humidity level on the surface of the leaves, a required condition for disease development. Disease severity level was scored on plant third leaves 23 days after the treatment (D23), corresponding to 21 days post-inoculation, by assessing leaf areas covered by lesions (chlorosis and necrosis) bearing or not pycnidia.

### *In planta* Fungal Staining Assay

The effect of Rh-Est-C12 on *in planta* spore germination and epiphytic fungal growth on leaf surface was examined on D5 (i.e., 3 days after inoculation) using the chitin dye Fluorescent Brightener 28 (Calcofluor; Sigma-Aldrich, Saint Louis, United States). Three-centimeter segments, harvested from third leaves of the wheat plants grown in the greenhouse, inoculated with *Z. tritici*, and treated or not with Rh-Est-C12 at 500 mg.L^−1^ as described above, were sampled. The segments were immersed for 5 min in a solution of 0.1% (w/v) Calcofluor with a 0.1-M Tris-HCl buffer at pH 8.5 before being rinsed twice in sterile osmosed water for 2 min and dried at room temperature. Leaf segments were then deposited on a glass slide, covered with a coverslip, and observed with an optic microscope (Eclipse 80i; Nikon, Champigny-sur-Marne, France) under UV light. Four germinated spore classes were assessed during notations under the microscope: class 1, non-germinated spore; class 2, germinated spore with a small germ tube; class 3, germinated spore with a developed germ tube; class 4, germinated spore with a strongly developed germ tube. Nine leaf segments, randomly selected from three different pots (three segments per pot), were used as replicates for each condition. The frequencies of spore classes were determined from the analyses of 100 distinct spores on each leaf segment (*n* = 900 spores). Pictures were obtained using a digital camera (DXM1200C; Nikon, Champigny-sur-Marne, France) coupled with the image capture software NIS-Elements BR (Nikon, Champigny-sur-Marne, France).

### RNA Extraction and Transcriptomics Analysis

Total RNAs from wheat leaves were extracted using the RNeasy Plant Mini Kit (Qiagen, Courtaboeuf, France). The quality of RNAs was measured with Nanodrop by analyzing their absorbance ratios, A260/280 and A260/230, which were found to range between 2 and 2.2. Besides, RNA quality was also examined with Bioanalyzer 2100 (Agilent, France), and a minimal RNA integrity number (RIN) of 8 was required for all the samples. A non-targeted gene expression analysis was performed using a Microarrays GE 4x44 chip purchased from Agilent (Santa Clara, CA, United States). The hybridization for all the conditions was carried out in triplicate, with each replicate generated using the total RNA extracted from three bulked leaves harvested from different pots (nine pots were used in total). All steps of RNA amplification, staining, hybridization, and washing were performed according to the manufacturer's indications. Slides were scanned at 5 μm/pixel resolution using a GenePix 4000B scanner (Molecular Devices Corporation, Sunnyvale, CA, United States), and the images were used for grid alignment and expression data digitization using the software GenePix Pro 6.0 (Molecular Devices Corporation, Sunnyvale, CA, United States). Transcriptomics data have been submitted to the NCBI GEO Archive for Functional Genomics Data with the accession number GSE178704.

### Metabolite Extraction and Analysis

Metabolite extraction was performed on ground freeze-dried wheat leaves (30–50 mg per sample) using 25 μl of methanol per mg dry weight. The extract was then incubated in an ultrasound bath for 10 min before centrifugation at 13,000 g at 10°C for 10 min. The supernatant was analyzed using a Dionex Ultimate 3000 UHPLC (Thermo Fisher Scientific, United States) system. Chromatographic separations were performed on a Nucleodur C18 HTec column (150 mm × 2 mm, 1.8 μm particle size; Macherey-Nagel, Germany) maintained at 30°C. The mobile phase consisted of acetonitrile/formic acid (0.1%, v/v, eluant A) and water/formic acid (0.1%, v/v, eluant B) at a flow rate of 0.3 ml.min^−1^. The gradient elution was programmed as follows: 0 to 1 min, 95% B; 1 to 2 min, 95 to 85% B; 2 to 7 min, 85 to 0% B; 7 to 9 min, 100% A. The sample volume injected was 1 μl. The UHPLC system was coupled to an Exactive Orbitrap (Thermo Fisher Scientific, United States) mass spectrometer equipped with an electrospray ionization (ESI) source operating in positive mode. Parameters were set at 300°C for ion transfer capillary temperature and 2,500 V for needle voltage. Nebulization with nitrogen sheath gas and auxiliary gas was maintained at 60 and 15 arbitrary units, respectively. Spectra were acquired within the m/z (mass-to-charge ratio) mass ranging from 100 to 1,000 atomic mass units (a.m.u.) using a resolution of 50,000 at m/z 200 a.m.u. The system was calibrated internally using dibutyl-phthalate as lock mass at m/z 279.1591, giving a mass accuracy lower than 1 ppm. The instruments were controlled using the Xcalibur software (Thermo Fisher Scientific, United States). LC-MS-grade methanol and acetonitrile were purchased from Roth Sochiel (France); water was provided by a Millipore water purification system. Apigenin and chloramphenicol (Sigma-Aldrich, France) were used as internal standards. Metabolomics data have been deposited to the EMBL-EBI MetaboLights database (Haug et al., 2020) with the identifier MTBLS4542.

### Statistical Analyses

The protection efficacy conferred by Rh-Est-C12 on wheat plants in the greenhouse was analyzed by one way analysis of variance (ANOVA) at *P* ≤ 0.05 using GraphPad Prism software version 9 (GraphPad Software Inc., San Diego, United States). *In planta* spore germination and hyphal growth were analyzed by Tukey's test at *P* ≤ 0.05 using GraphPad Prism software version 9. Half-maximal inhibitory concentration (IC_50_) was calculated from the dose-response curve of Rh-Est-C12 using GraphPad Prism software version 9.

Gene expression data of the transcriptomic assay were normalized with the Quantile algorithm. The three control samples were filtered for *P* < 0.05, and the average was calculated for each gene. A fold change value was calculated for each gene by comparing samples to the mean of corresponding control. Differentially expressed genes (DEGs) were selected for a significant threshold >2 or < 0.5 (*P* < 0.05). Functional annotations of the DEGs were based on NCBI GenBank, and related-genes physiological processes were assigned with NCBI, AmiGO 2 Gene Ontology, and UniProt. A Kyoto Encyclopedia of Genes and Genomes (KEGG) pathway analysis was also conducted to identify relevant biological pathways for the selected genes. However, DEGs may be involved in more than one biological process, especially when it comes to stress responses; hence, the determined functional annotation could be subjected to modifications in the future.

For metabolomic analyses, metabolite selection and identification were based on previously published studies on benzoxazinoids (de Bruijn et al., 2016), flavonoids (Wojakowska et al., 2013), and hydroxycinnamic acid amides (Li et al., 2018) from wheat. Besides, the proposed putative metabolite identifications were based on expertized analysis of corresponding mass spectra and comparison with published reports in the literature. Additional information was retrieved from the KEGG and PubChem databases. A relative quantification of the selected metabolites was performed using the software Xcalibur. The identity of some metabolites was confirmed using the corresponding standards provided by Sigma-Aldrich (France). Differential metabolomic analyses among the different conditions were performed using Tukey's honest significant difference method followed by false discovery rate (FDR) correction using the Benjamini-Hochberg procedure (Benjamini and Hochberg, 1995). Metabolites of interest were considered differentially accumulated when the false discovery rate was below 5 % (FDR < 0.05).

## Results

### Rh-Est-C12 Displays Direct Antifungal Activity and Reduces the Severity of Disease Caused by *Z. tritici*

The Rh-Est-C12 (Figure 1A)-induced protection in wheat against *Z. tritici* was assessed in the greenhouse (Figure 1B). Twenty-three days after the treatment (D23), i.e., 21 days after inoculation, the disease severity scored on the third leaves of the non-treated inoculated control plants consisted of 55.7% of diseased leaf area. The preventive Rh-Est-C12 treatment applied at 500 mg.L^−1^ 2 days before inoculation resulted in significant reduction of the disease symptoms caused by the pathogen, with only 32.8% of diseased leaf area on the third leaves, corresponding to a reduction of 41.1% (Figure 1B). No phytotoxic effect (neither chlorotic and necrotic lesions nor impact on plant development) was visually observed on wheat leaves at the tested concentration. Additionally, *in vitro* bioassays were performed to assess the direct antifungal effect of Rh-Est-C12 on the fungus. The dose-response curve revealed total inhibition of *in vitro* fungal growth with a concentration of 31.2 mg.L^−1^ and with an IC_50_ value of 26.8 mg.L^−1^ (Figures 1C,D). Finally, *in planta* cytological staining was carried out to examine the effect of RL on *Z. tritici* early infection process. The results revealed that Rh-Est-C12 impacts on D5 (five days after treatment) in both spore germination and epiphytic hyphal growth of the fungus. The proportion of each class of spores was significantly different between Rh-Est-C12-treated and control plants (Figure 1E). In the control plants on D5, *Z tritici* spores were mainly germinated with a developed or strongly developed germ tube (classes 3 and 4), while in the Rh-Est-C12-treated plants, *Z. tritici* spores were less developed and were mostly present as non-germinated spores or germinated spores with only a small germ tube (classes 1 and 2) (Figure 1E).

### Rh-Est-C12 Does Not Induce Major Modifications in Wheat Leaf Gene Expression

An RNA microarray chip assay was performed to investigate the effect of Rh-Est-C12 on the wheat transcriptome in the absence or presence of *Z. tritici* 2 days after the treatment (D2) under noninfectious conditions, and 5 days after the treatment (D5), i.e., 3 days after inoculation, under both noninfectious and infectious conditions. These two time points were chosen, because they correspond to the early stages of both plant defense mechanism induction (D2) and *Z. tritici* infection (D5). The eliciting potential of Rh-Est-C12 was assessed on D2 and D5 by comparing treated and non-treated plants under non-infectious conditions. The priming of plant defenses was evaluated on D5 by comparing treated and non-treated plants under infectious conditions (treated and inoculated plants *vs*. non-treated inoculated plants), and by taking into account the potential elicitation effect of Rh-Est-C12 on D2 and D5. Moreover, additional comparisons were performed (treated and inoculated plants *vs*. treated non-inoculated plants and treated and inoculated plants *vs*. non-treated non-inoculated plants) to get a deeper understanding of the potential priming effect of Rh-Est-C12 on wheat plants (Supplementary Table S1 and Supplementary Figures S1, S2). Thereafter, the different comparisons will be referred to as eliciting, fungal, and priming effects, corresponding to elicitation, fungal infection alone, and priming modalities, respectively.

**Figure 2:**
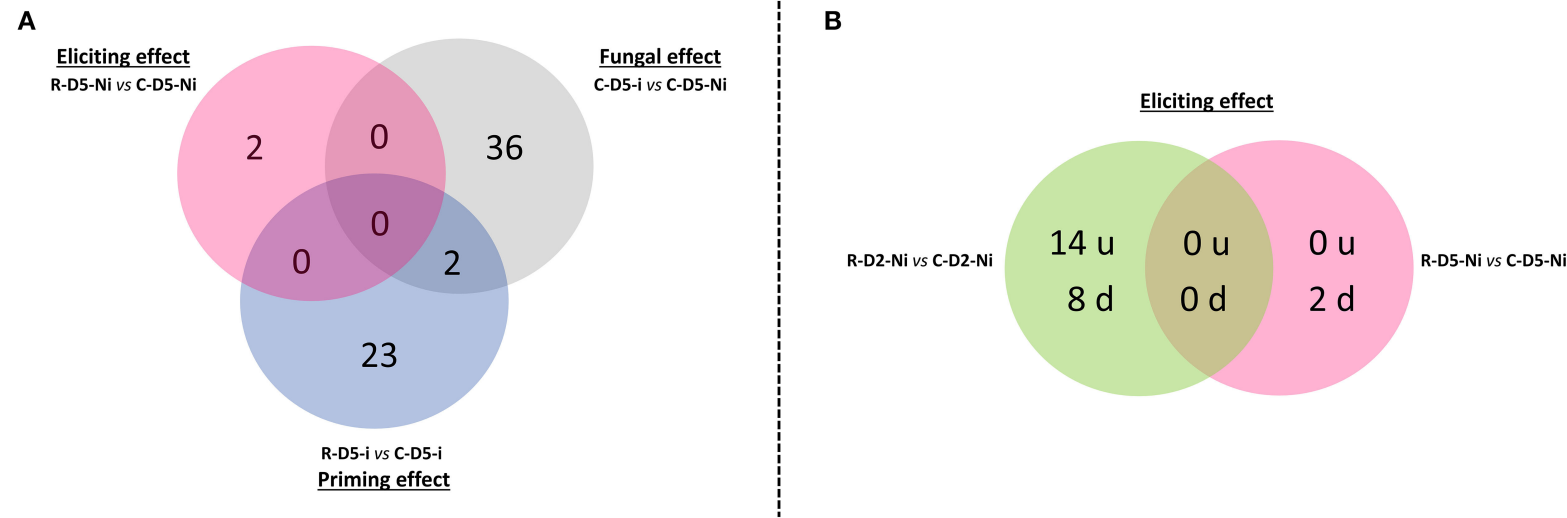
Venn diagrams of the number of differentially expressed genes observed during Rh-Est-C12 eliciting and priming, as well as *Z. tritici* effects. **(A)** The eliciting (R-D5-Ni *vs*. C-D5-Ni), fungal (C-D5-i *vs*. C-D5-Ni), and priming effects (R-D5-i *vs*. C-D5-i) on the number of differentially expressed genes in wheat third leaves (Cv. Alixan) were compared at 5 days after treatment with Rh-Est-C12, *i.e*. at 3 days after infection with Z. tritici (T02596 strain). **(B)** The effect of the rhamnolipid alone, applied at 500 mg.L^−1^, on the number of upregulated (u) and downregulated (d) genes was explored 2 and 5 days after treatment (R-D2-Ni *vs*. C-D2-Ni and R-D5-Ni *vs*. C-D5-Ni, respectively). C stands for a mock treatment while R is for Rh-Est-C12 application; Ni relates to mock-inoculated wheat, while i means inoculated plants with *Z. tritici*; D2 or D5 indicates sampling dates of the third-leaves corresponding to 2 or 5 days after treatment, respectively.

**Figure 3:**
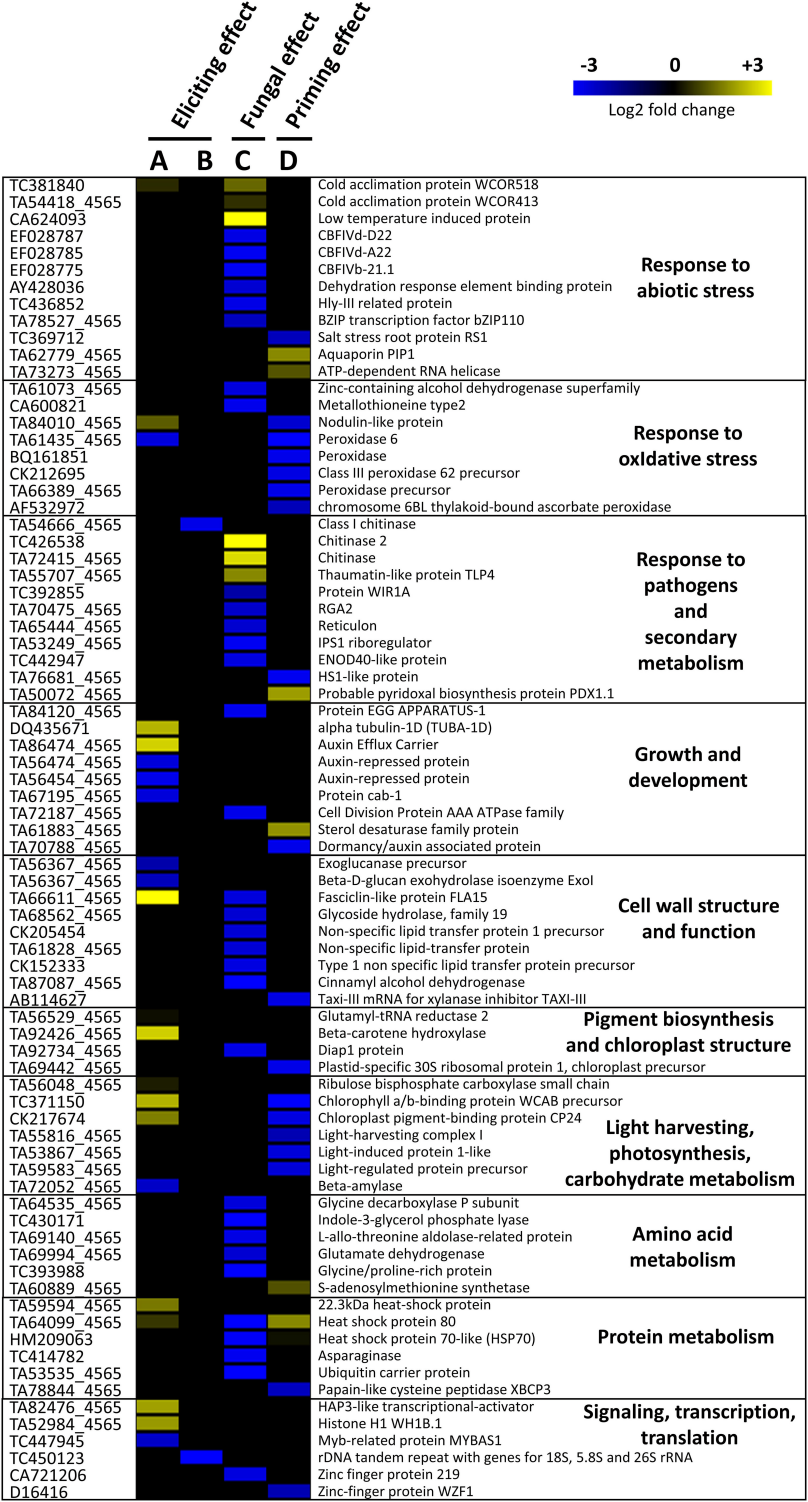
Heatmap representation of differentially expressed genes in wheat leaves (Cv. Alixan) in response to foliar application of 500 mg.L^−1^ Rh-Est-C12 and/or inoculation with *Z. tritici* (T02596 strain) **(A)** 2 and **(B–D)** 5 days after treatment. Columns **(A)** and **(B)** illustrate the effect of the rhamnolipid without further inoculation with the fungus, i.e., the eliciting effect, respectively, 2 (R-D2-Ni *vs*. C-D2-Ni) and 5 days (R-D5-Ni *vs*. C-D5-Ni) after treatment. **(C)** Represents the fungal effect alone (C-D5-i *vs*. C-D5-Ni), while **(D)** shows the potential priming activity of Rh-Est-C12 (R-D5-i *vs*. C-D5-i). Gene-related physiological processes are represented on the right part of the heatmap and were determined using NCBI, AmiGO 2 Gene Ontology, KEGG, and UniProt. Significant relative change in gene transcription is expressed in the Log2 ratio, according to the yellow-blue color scale using the WebMev software. C stands for a mock treatment, while R is for Rh-Est-C12 application; Ni relates to mock-inoculated wheat, while i means inoculated plants with *Z. tritici*; D2 or D5 indicates sampling dates of the third leaves, corresponding to 2 or 5 days after treatment, respectively.

Overall, out of the 43,803 wheat probes available in the RNA microarray chip, 78 differentially expressed genes (DEGs) were highlighted when taking into account all the studied modalities, performed comparisons, and all time points (Supplementary Table S2). Overall, only a weak effect on gene expression was observed in both the elicitation (24 DEGs on D2 and D5) and priming (25 DEGs on D5) modalities when compared to the transcriptional effect of the fungal infection alone (38 DEGs, D5) (Figure 2A). Only two DEGs common between the priming and fungal effect modalities were identified at D5 (Figure 2A). Interestingly, most of the changes observed in gene expression profiles correspond to downregulations, except on D2 in the eliciting modality where a higher number of upregulated genes was found when compared to downregulated ones (Figures 2B, 3).

**Figure 4:**
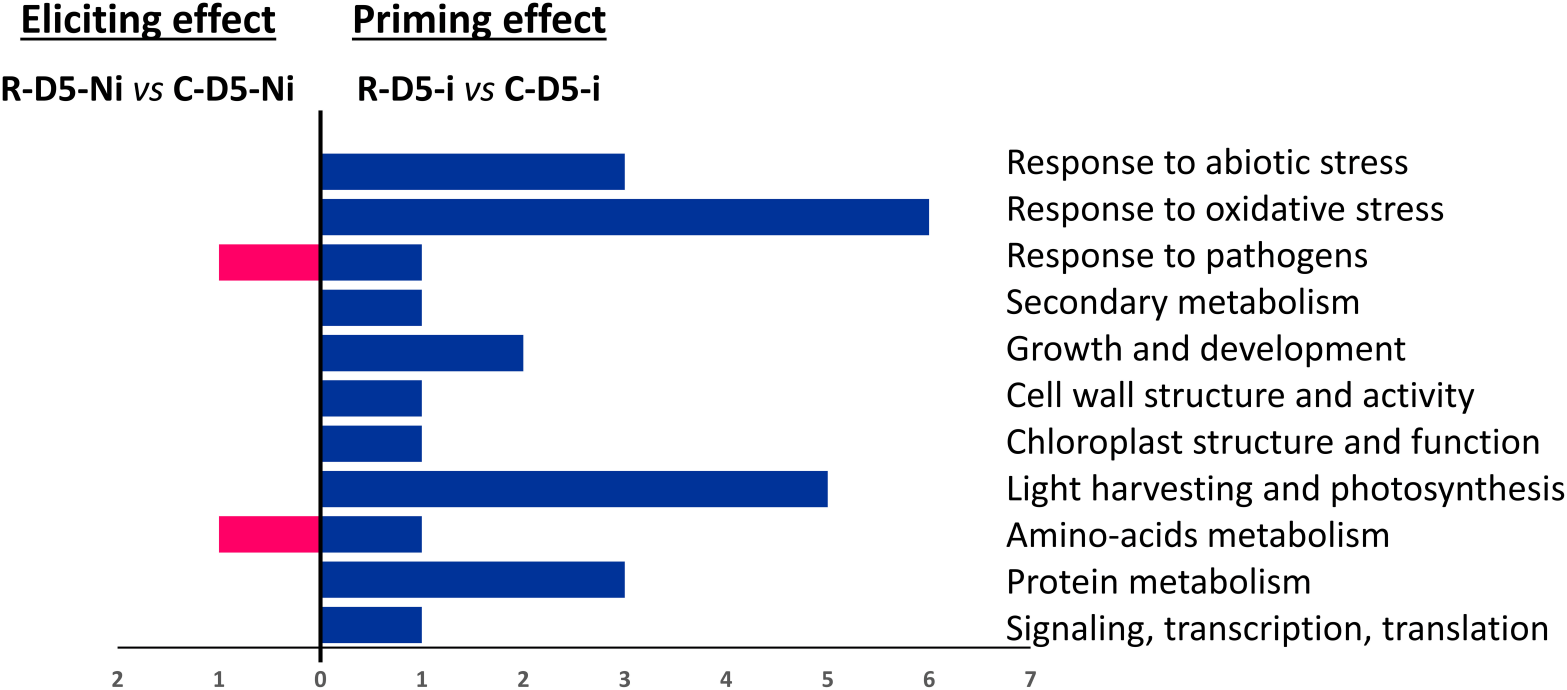
Functional groups of differentially expressed genes (DEGs) observed in eliciting (R-D5-Ni *vs*. C-D5-Ni) compared to priming (R-D5-i *vs*. C-D5-i) conditions. DEGs were classified into different functional groups according to NCBI GenBank. C stands for a mock treatment, while R is for Rh-Est-C12 application; Ni relates to mock-inoculated wheat while i means inoculated plants with *Z. tritici*; D5 indicates the sampling date of the third leaves, corresponding to 5 days after treatment.

When considering the eliciting modality, on D5, almost no transcriptional effect was observed in the treated wheat plants. We could notice a downregulation of the expression of two DEGs, one encoding a class 1 chitinase and the other encoding an “rDNA tandem repeat with genes for 18S, 5.8S, and 26S rRNAs” (Figure 3). However, in an earlier post-treatment stage (D2), 22 DEGs were found. These genes encode for proteins involved in responses to abiotic and oxidative stresses, growth and development, cell-wall structure and function, pigment biosynthesis and chloroplast structure, light-harvesting, photosynthesis and carbohydrate metabolism, protein metabolism, signaling transcription, and translation (Figure 4). Regarding the priming modality on D5, among the 25 DEGs highlighted, only seven were up-regulated, while 18 were down-regulated. All of these DEGs were different from the ones found in the eliciting and fungal effect modalities, except for two DEGs encoding for heat shock protein 80 and heat shock protein 70-like, which were also deregulated in the fungal effect modality (Figures 2A, 3). Genes regulated in the priming modality encode for proteins involved in diverse functions and belong to all targeted functional groups of genes (Figure 4). Finally, the fungal infection alone displayed the most important effect on the wheat leaf transcriptome when compared to the eliciting and priming modalities. Again, most of the changes observed were due to gene downregulations (six genes upregulated *vs*. 32 genes downregulated). All functional groups of genes were represented in this modality, except for the gene functional groups light-harvesting, photosynthesis, and carbohydrate metabolism (Figure 3).

### Rh-Est-C12 Does Not Cause Marked Changes in Wheat Leaf Metabolite Accumulation

**Figure 5:**
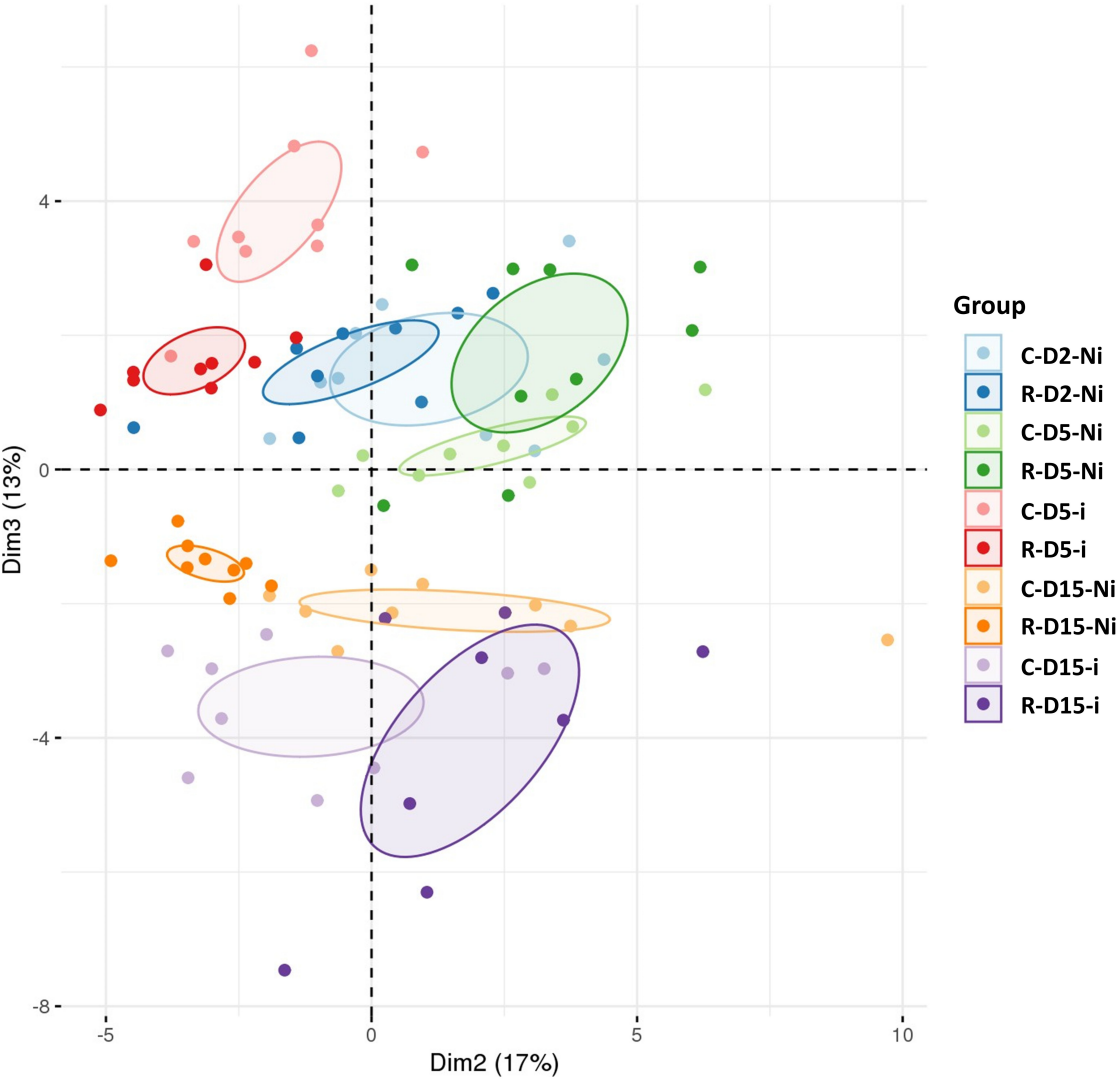
Principal component analysis (PCA) of targeted metabolomic data sets from wheat leaves treated or not with Rh-Est-C12 and/or inoculated with *Z. tritici*. For each modality, nine biological replicates were used. The second and the third principal components explain 17 and 13% of the variance separating the groups of samples, respectively. C stands for a mock treatment, while R is for Rh-Est-C12 application; Ni relates to mock-inoculated wheat, while i means inoculated plants with *Z. tritici*; D2, D5 or D15 indicates sampling dates of the third leaves, corresponding to 2, 5, or 15 days after treatment, respectively.

A UHPLC-MS analysis was carried out to examine the effect of Rh-Est-C12 on the wheat leaf metabolome, at the same time points as those selected for transcriptomic analysis (D2 and D5) and at an additional time point at 15 days after treatment (D15), i.e., 13 days after inoculation. This time point corresponds to the moment preceding the switch of the fungus from the biotrophic to the necrotrophic phase. All comparisons aiming at investigating the eliciting, priming, and fungal effects were undertaken according to the same pairwise comparison performed above in the transcriptomics assay. A total of 54 metabolites belonging to different chemical families were identified, and their relative amounts were determined for all the performed comparisons at all time points (Supplementary Table S3). PCA analysis revealed marked clustering of the treated and/or inoculated plants, indicating that Rh-Est-C12 and/or *Z. tritici* affects wheat metabolite patterns (Figure 5).

**Figure 6:**
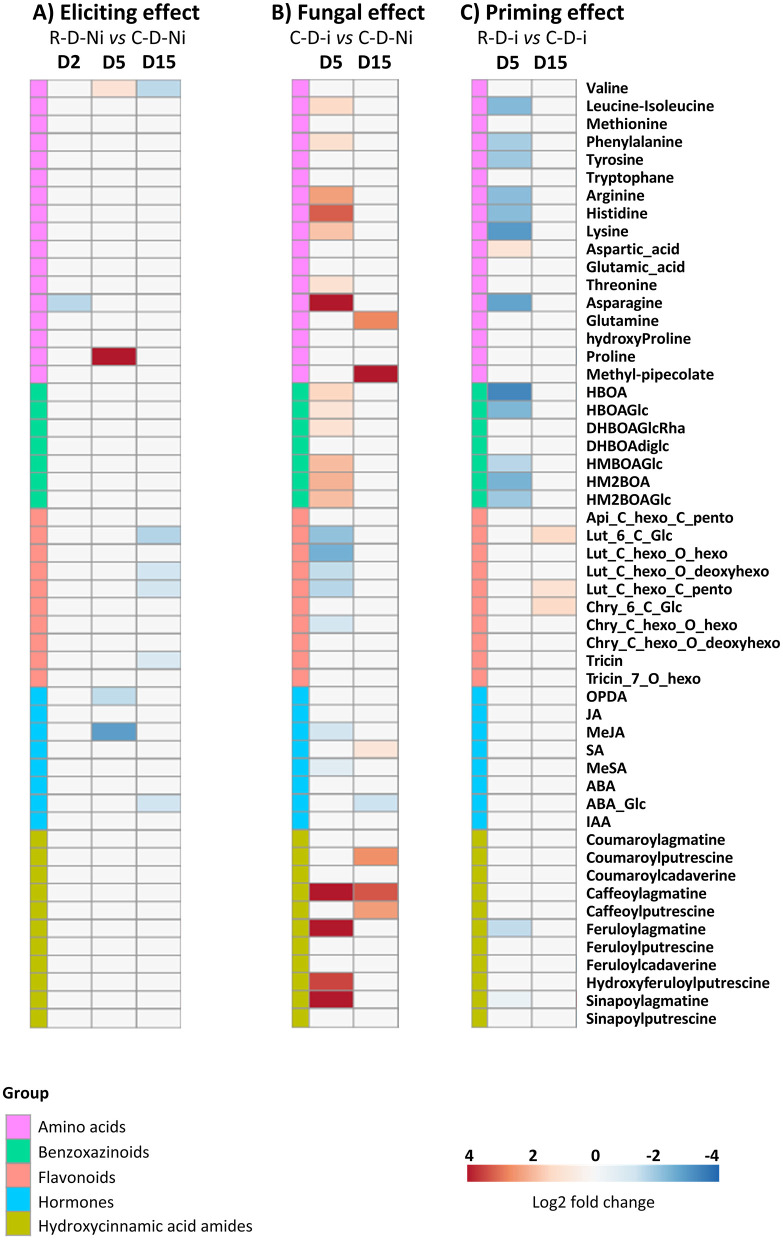
Heatmap representation of differentially accumulated metabolites in wheat third leaves (Cv. Alixan) treated or not with Rh-Est-C12 and infected or not with *Z. tritici* (T02596 strain) at different time points. With metabolite relative accumulation changes in the **(A)** eliciting modalities, from left to right, R-D2-Ni *vs*. C-D2-Ni, R-D5-Ni *vs*. C-D5-Ni, and R-D15-Ni *vs*. C-D15-Ni; **(B)** fungal modalities, C-D5-i *vs*. C-D5-Ni and C-D15-i *vs*. C-D15-Ni; **(C)** priming modalities, R-D5-i *vs*. C-D5-i and R-D15-i *vs*. C-D15-i. Log2 of significant metabolite fold changes for indicated pairwise comparisons are given by shades of red or blue colors according to the corresponding scale bar. Metabolites were grouped according to their functional or chemical family as amino acids, benzoxazinoids, flavonoids, hormones, or hydroxycinnamic acid amides. Data represent the mean values of nine biological replicates for each condition and time point. Statistical analyses were performed by Tukey's honest significant difference method followed by false discovery rate (FDR) correction, with FDR <0.05. For FDR ≥0.05, Log2 fold changes were set to 0. Abbreviations: C stands for a mock treatment, while R is for Rh-Est-C12 application; Ni relates to mock-inoculated wheat, while i means inoculated plants with *Z. tritici*; D2, D5, or D15 indicates sampling dates of the third leaves, corresponding to 2, 5, or 15 days after treatment, respectively.

When focusing on the eliciting modality, only a few differentially accumulated metabolites (DAMs) in the treated non-inoculated leaves were highlighted at the three targeted time points (Figure 6). On D2, one amino acid (asparagine) was found depleted, whereas on D5, the concentrations of two phytohormones and precursors (MeJA and OPDA) were significantly decreased. Moreover, two amino acids (valine and proline) were enriched at this later time-point. On D15, six metabolites were significantly depleted, one amino acid (valine), one phytohormone, abscisic acid glucose ester (ABA-Glc), and four flavonoids, luteolin-6-C-hexoside (luteolin-6-C-Glc), luteolin-C-hexosyl-deoxyhexoside (luteolin-C-hexo-O-deoxyhexo), luteolin-C-hexosyl-C-pentoside (luteolin-C-hexo-C-pento), and tricin. In the priming modality, more DAMs were observed than in the eliciting modality but on D5 only. On D5, 13 DAMs were depleted, seven of them were amino acids, leucine-isoleucine, phenylalanine, tyrosine, arginine, histidine, lysine, and asparagine; five were benzoxazinoids, HBOA, HBOA-glucoside (HBOAGlc), HMBOA-hexoside (HMBOAGlc), HM2BOA, and HM2BOA-hexoside (HM2BOAGlc); one is a hydroxycinnamic acid amide, feruloylagmatine. It should be noticed that most of these depleted DAMs were identical to those that were enriched in the fungal effect modality. The enrichment of only one metabolite, the amino acid aspartic acid, can be associated with the priming modality. No DAMs belonging to the flavonoid and phytohormone families were highlighted at this time point. On D15, however, an increase in the concentration of three flavonoids, luteolin-6-C-Glc, luteolin-C-hexo-C-pento, and chrysoeriol-6-C-hexoside (chrysoeriol-6-C-Glc), was observed. Fungal infection alone caused more changes in leaf metabolite concentrations than the eliciting and priming modalities. Indeed, on D5, among the 54 identified metabolites, 24 were differently accumulated (17 enriched, seven depleted) in the fungal alone modality. On D15, considerably less modifications were detected, with only seven DAMs, among them six were enriched and one was depleted (Figure 6).

## Discussion

Here, we combined transcriptomic and metabolomic approaches to explore the ability of a bioinspired mono-RL (Rh-Est-C12) to trigger defense reactions in wheat toward *Z. tritici*, a significantly damaging pathogen of this major agroeconomic crop. The results confirmed that Rh-Est-C12 significantly protects wheat against the pathogen by reducing disease severity by almost half and by displaying a direct antimicrobial activity toward the fungus both *in vitro* and *in planta*, thus agreeing with a previous report testing the potential activity of this RL, as well as other RLs with different structures, in the wheat-*Z. tritici* pathosystem (Platel et al., 2021). The antimicrobial activity of RLs against phytopathogens, including fungi and oomycetes, has been reported in many studies, as reviewed recently by Crouzet et al. (2020). However, studies investigating RL-induced plant resistance using omics approaches are still lacking. Our results reveal that Rh-Est-C12 does not induce major modifications in either the patterns of gene expression or metabolite accumulation when considering both the eliciting and priming modalities.

### Rh-Est-C12 Alone Triggers Only Slight Alterations of Wheat Leaf Metabolism

Among the DEGs detected in the eliciting modality, a much higher number (22) was scored on D2 than on D5 (2), suggesting that the slight eliciting effect observed with Rh-Est-C12 at the earlier time point D2 is not persistent and seems to disappear 3 days later on D5. Out of the 22 detected DEGs, two encode heat shock proteins (HSPs), 22.3kDa heat-shock protein, and heat shock protein 80. These chaperone proteins are crucial molecules involved in plant defense against abiotic and biotic stresses. As reviewed by ul Haq et al. (2019), HSPs may increase ROS scavenging by enhancing plant antioxidant enzymes. Interestingly, we have previously found that Rh-Est-C12 increased catalase and peroxidase activities in wheat (Platel et al., 2021). Besides, HSPs are also considered to play a role in membrane stability (ul Haq et al., 2019). Remarkably, RLs are supposed to inhibit microbial pathogens and trigger plant immunity defenses by interacting directly with their plasma membranes (Crouzet et al., 2020). Hence, the accumulation of HSP transcripts could suggest that Rh-Est-C12 can affect wheat-cell membrane stability by mimicking abiotic stress. This hypothesis is supported by the deregulation of abiotic stress response-associated DEGs observed in this modality, such as those coding for cold acclimation protein WCOR518, fasciclin-like protein FLA15, β-carotene hydroxylase, peroxidase 6, β-amylase, and myb-related protein MYBAS1 (Davison et al., 2002; Kaplan and Guy, 2004; Svensson et al., 2006; You and Chan, 2015; Zang et al., 2015; Peixoto-Junior et al., 2018). At the metabolome level, we, on D5 the enrichment of two amino acids, valine and proline, and the depletion of two phytohormones and precursors, MeJA and OPDA. Proline is an osmolyte metabolite involved in adaptation and tolerance to a large variety of abiotic stresses, such as osmotic, freezing, and cold stresses (Szabados and Savour, 2010; Lv et al., 2011). The accumulation of this metabolite could likely be a consequence of the Rh-Est-C12 “abiotic stress-like” effect on wheat. Also, on D15, the concentration of only a few DAMs was altered. Taken as a whole, these results suggest that Rh-Est-C12 treatment, in the elicitation modality, triggers some modifications in the wheat plant transcriptome and metabolome, but that its effect is limited, especially when compared to the deregulation levels induced by other elicitors in other plants. For instance, Landi et al. (2017) recently reported by RNA-seq that preharvest treatment of strawberry fruits with chitosan and BTH induced modifications in the expression of 5,062 and 5,210 transcripts, respectively, 6, 12, and 24 h post-treatment compared to control conditions. In grapevine, plant treatment with the beta-glucan laminarin and its sulfated derivative (PS3) induced the regulation of 94 and 132 genes 12 h post-treatment, respectively, highlighted (Gauthier et al., 2014) using a microarray chip. The fact that Rh-Est-C12 induces only slight alterations on the plant transcriptome and metabolome, even with the relatively high concentration used (500 mg.L^−1^), indicates that this RL could provide protection to wheat against *Z. tritici* without high fitness cost on the plant. Such a positive property is sought after for crop protection compounds, since yield should not be negatively impacted by compounds having a insignificant impact on plant physiology (Cipollini et al., 2017). Conversely, elicitors displaying a large extent of alterations in host physiology, as those presented above, may lead to protection but with possibly higher allocation costs of the plant (Tripathi et al., 2019). The suggested neutral effect of Rh-Est-C12 on wheat fitness cost should furthermore be ascertained with appropriate assays taking into account, for instance, wheat grain filling and yield.

### Rh-Est-C12 Does Not Prime Strong Defense Responses in Wheat Against *Z. tritici*

In the modality assessing the potential priming effect of Rh-Est-C12, we detected a significant downregulation of 18 genes on D5, among them six were involved in response to oxidative stress (encoding mainly for peroxidases) and five were related to light-harvesting, photosynthesis, and carbohydrate metabolism (encoding for chlorophyll a/b-binding protein WCAB precursor, chloroplast pigment-binding protein CP24, light-harvesting complex I, light-induced protein 1-like, and light-regulated protein precursor). The other downregulated DEGs were associated with other functions. Two of these DEGs code for proteins particularly interesting in plant defense against pathogens, (i) the xylanase inhibitor TAXI-III that was reported as being potentially involved in durum wheat resistance against *Fusarium graminearum*, and (ii) the papain-like cysteine peptidase, a central key-enzyme in plant immunity (Moscetti et al., 2013; Misas-Villamil et al., 2016). Additionally, six DEGs were over-expressed (genes encoding for aquaporin PIP1, ATP-dependent RNA helicase, probable pyridoxal biosynthesis protein PDX1.1, sterol desaturase family protein, S-adenosylmethionine synthetase, and heat-shock protein 80). Among these genes, some may be involved in plant defense against biotic stress, like those encoding for aquaporin PIP1 and ATP-dependent RNA helicase (Zhang et al., 2014; Li et al., 2020). Remarkably, out of all the 25 detected DEGs in the priming modality, only the ones encoding for heat-shock protein 80 and heat shock protein 70-like were found in another condition (*Z. tritici* infection alone) on D5. In addition, the effect of RL on the priming modality seems weak, with only a few of the detected DEGs thought to be involved in plant resistance to pathogens. On the wheat metabolome, also, only a few changes were observed. On D5, aspartic acid, which is known to be involved in response to abiotic stress (Han et al., 2021), was the only metabolite significantly enriched, whereas 13 DAMs were depleted. Most of them, however, were found enriched in the fungal effect modality and totally absent in the comparison treated-inoculated *vs*. non-treated non-inoculated (Supplementary Figure S3), suggesting that the observed metabolite depletions in the priming modality on D5 are not conferred by the RL treatment but rather by the reduced fungal infection only. Nevertheless, a late enrichment of three flavonoids was observed on D15 in the treated and inoculated plants, suggesting a potential enhancement of plant responses to the pathogen at this time point. Indeed, flavonoids are secondary metabolites biosynthesized from the phenylpropanoid pathway and have been described as antimicrobial and antioxidant compounds that could be involved in wheat defense against biotic stress (Pietta, 2000; Mierziak et al., 2014).

### *Z. tritici* Infection Induces Significant Modifications in Wheat Leaf Transcriptome and Metabolome

In addition to the analysis of Rh-Est-C12 biological activity, our study provides insights regarding the effect of *Z. tritici* alone on the wheat transcriptome and metabolome in the case of a compatible interaction. On D5, we observed alterations in the accumulation of transcripts of 43 genes (with all functional groups represented, except for light-harvesting, photosynthesis, and carbohydrate metabolism) and 24metabolites (all functional or chemical families represented) corresponding to 44% of all the targeted metabolites, much more than in the other tested modalities (i.e., elicitation and priming ones). The regulation of these DEGs and DAMs may be caused by (i) plant recognition of the fungus and (ii) subsequent defense response deployment, i.e., upregulation of genes encoding for pathogenesis-related (PR) proteins, chitinases, and thaumatin-like proteins, as well as over-accumulation of specific amino acids, benzoxazinoids, and hydroxycinnamic acid amides. Noteworthy is that PR-proteins are key components of the immune system of plants (Ali et al., 2018). Benzoxazinoids are plant defense metabolites found in many *Poaceae* species and are known to display antimicrobial and allelopathic activities, as well as to regulate callose deposition, like in maize (Hashimoto and Shudo, 1996; de Bruijn et al., 2018). Hydroxycinnamic acid amides are another family of plant secondary metabolites reported to be positively correlated with plant resistance in several pathosystems (Muroi et al., 2009; Gunnaiah et al., 2012; Yogendra et al., 2014). Seybold et al. (2020) described significant modifications in the accumulation of benzoxazinoids and hydroxycinnamic acid amides during Z. tritici infection, between susceptible and resistant cultivars, underlining the potential importance of these metabolites in wheat resistance to the pathogen. Interestingly, we did not observe any significant accumulation of benzoxazinoids on D15. This result may be associated with the observed downregulation of the gene coding forindole-3-glycerol phosphate lyase on D5. This enzyme is known to catalyze indole formation, a precursor of the benzoxazinoid biosynthesis pathway (Gierl and Frey, 2001). Seybold et al. (2020) also observed an inhibition of benzoxazinoid accumulation in a susceptible wheat cultivar (Obelisk) in the early stage of *Z. tritici* infection only, hypothetically explained by fungal effector secretion and host defense manipulation by the pathogen during the studied interaction.

Another possible explanation of the observed modification of DEGs and DAMs in wheat leaves during the infection alone is the “hijacking” of plant metabolism by the fungus to facilitate its invasion. Indeed, still on D5, 37 out of the 43 detected DEGs were downregulated. These genes were particularly associated with response to stress (abiotic, oxidative, and pathogens) and secondary metabolism, as well as cell-wall structure and function and amino acid and protein metabolism, suggesting a deleterious effect of the fungus on these functions. In particular, we observed a significant downregulation of genes coding for HSPs, key proteins involved in plant response to different stresses (ul Haq et al., 2019). We also recorded a downregulation of genes coding for proteins WIR1A and RGA2, two genes likely involved in wheat resistance against other fungal diseases, such as stripe and leaf rust (Loutre et al., 2009; Coram et al., 2010). Our results also suggest that *Z. tritici* could impair cell-wall strengthening in the studied Alixan wheat cultivar. Indeed, the fungal infection induced downregulation of six DEGs associated with cell wall structure and function, including genes encoding for cinnamyl alcohol dehydrogenase and non-specific lipid transfer proteins and precursors. Cinnamyl alcohol dehydrogenase is a key enzyme of the lignin biosynthesis pathway, for which encoding genes were also found downregulated by Rudd et al. (2015) on wheat 1 day after infection with *Z. tritici*. Seybold et al. (2020) showed lignin under-accumulation in a susceptible cultivar (Obelisk) compared to a resistant one (Chinese spring), suggesting the role of this crucial monolignol polymer in wheat resistance to *Z. tritici*. Moreover, nonspecific lipid transfer proteins (nsLTPs) also play critical roles in protective mechanisms, such as cell-wall organization, against pathogens and have even been classified as part of the PR-14 family. The other functions of this protein family, such as antimicrobial activity and systemic acquired resistance (SAR) signaling, in plant response to biotic stress have been reviewed by Liu et al. (2015). Moreover, we observed on D15 the depletion of five flavonoids. Flavonoids are known to accumulate in cell walls during infection (Mierziak et al., 2014). A decrease in their concentration may be caused by overcoming of wheat defense reactions by the fungus. In addition, we observed significant depletion of MeJA and MeSA on D5, suggesting repression of the jasmonic acid (JA) and salicylic acid (SA) pathways at this time point. These phytohormones are particularly crucial in defense against pathogen attacks. Usually, SA is considered to be the key hormone regulating defense against biotrophic pathogens, while JA would trigger defense mechanisms toward necrotrophic invaders. MeSA, a methyl form of SA, is also known as a key component in SAR onset (Thaler et al., 2012; Chen et al., 2019). In the wheat-*Z. tritici* pathosystem, Rudd et al. (2015) also detected downregulation of the SA and JA pathways at the beginning of the *Z. tritici* biotrophic phase in the susceptible cultivar Riband. The authors also reported SA accumulation during the fungal necrotrophic phase, whereas we observed a significant increase in SA concentration in wheat leaves on D15, i.e., at the end of the biotrophic phase, suggesting that SA regulation during *Z. tritici* infection is cultivar-dependent.

## Conclusion

Although Rh-Est-C12 treatment triggers significant regulations, especially on wheat leaf gene expression on D2, possibly caused by “abiotic stress-like” response due to its potential interaction with plant plasma membranes, it appears in our conditions that the RL protects wheat against *Z. tritici* without causing major alterations in plant metabolism. Our results suggest that (i) Rh-Est-C12 likely displays a neutral fitness cost in the plant at the tested concentration and (ii) that the molecule-induced protection is mainly due to its direct antifungal activity against the pathogen and, to a lesser extent, to induction of plant defenses. This study also provides new insights into molecular bases of the wheat-*Z. tritici* interaction.

## Data Availability Statement

The datasets presented in this study can be found in online repositories. The names of the repository/repositories and accession number(s) can be found in the article/Supplementary Material.

## Author Contributions

RP, MM-R, BR, PT, PHa, PM, J-LH, MH, PHu, PR, and AS conceived the project. RP, AL-D, RB, AM-G, LC, MM-R, BR, PT, PHa, PM, J-LH, MH, PHu, PR, and AS designed and/or performed the experiments. RP, AL-D, RB, AM-G, LC, PHu, and AS collected and analyzed the data. RP and AS wrote the first draft of the manuscript. All the authors contributed to the manuscript and approved the submitted version.

## Funding

This research was conducted in the framework of the project Bioscreen (Smartbiocontrol portfolio), funded by the France-Wallonie-Vlaanderen European program, Interreg V, and the CPER Alibiotech, funded by the European Union, the French State, and the French Council Hauts-de-France. Part of the publication fees of this article was supported by the Catholic University of Lille, France.

## Conflict of Interest

The authors declare that the research was conducted in the absence of any commercial or financial relationships that could be construed as a potential conflict of interest.

## Publisher's Note

All claims expressed in this article are solely those of the authors and do not necessarily represent those of their affiliated organizations, or those of the publisher, the editors and the reviewers. Any product that may be evaluated in this article, or claim that may be made by its manufacturer, is not guaranteed or endorsed by the publisher.
