# Supplementary material for: Bioinspired Rhamnolipid Protects Wheat Against Zymoseptoria tritici Through Mainly Direct Antifungal Activity and Without Major Impact on Leaf Physiology
Source: Front Plant Sci. 2022 Jun 3;13:878272. doi: 10.3389/fpls.2022.878272 (PMC9204090; doi:10.3389/fpls.2022.878272)
Supplement: Supplementary file 1 [file Data_Sheet_1.PDF]

SUPPLEMENTARY FIGURES

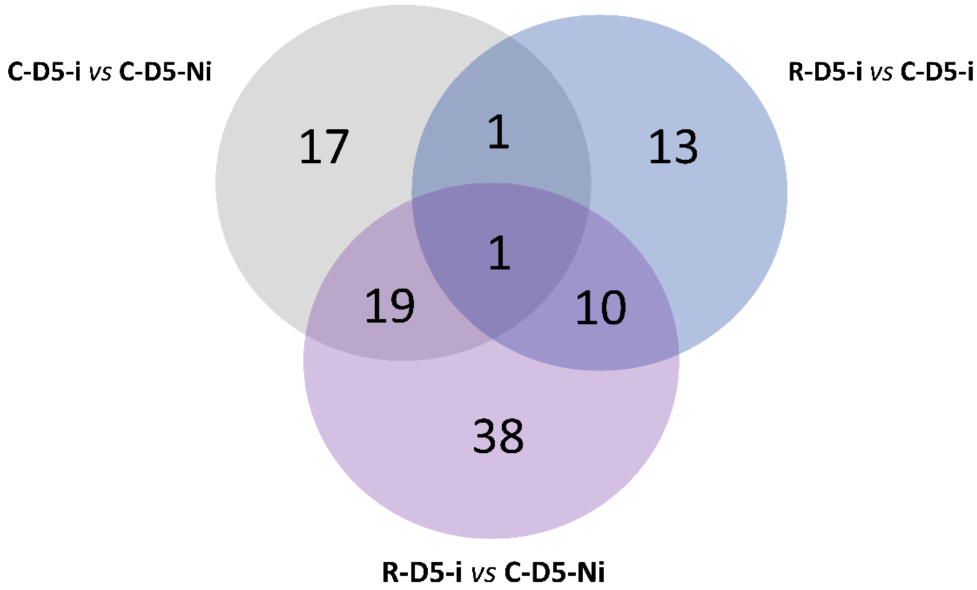

SUPPLEMENTARY FIGURE 1 | Venn diagram of the number of differentially expressed genes observed during the fungus effect (C-D5-i *vs* C-D5-Ni), the priming effect (R-D5-i vs C-D5-i) and the whole deregulation on gene expression caused by the rhamnolipid treatment followed by *Z. tritici* infection (R-D5-i *vs* C-D5-Ni) at five days after treatment. Wheat cultivar Alixan, *Z. tritici* T02596 strain and a rhamnolipid concentration of 500 mg.L^{-1} were used. Abbreviations : C stands for a mock treatment while R is for Rh-Est-C12 application ; Ni relates to a mock inoculated wheat while i means inoculated plants with *Z. tritici* ; D5 indicates the sampling date of the third-leaves, corresponding to five days after treatment.

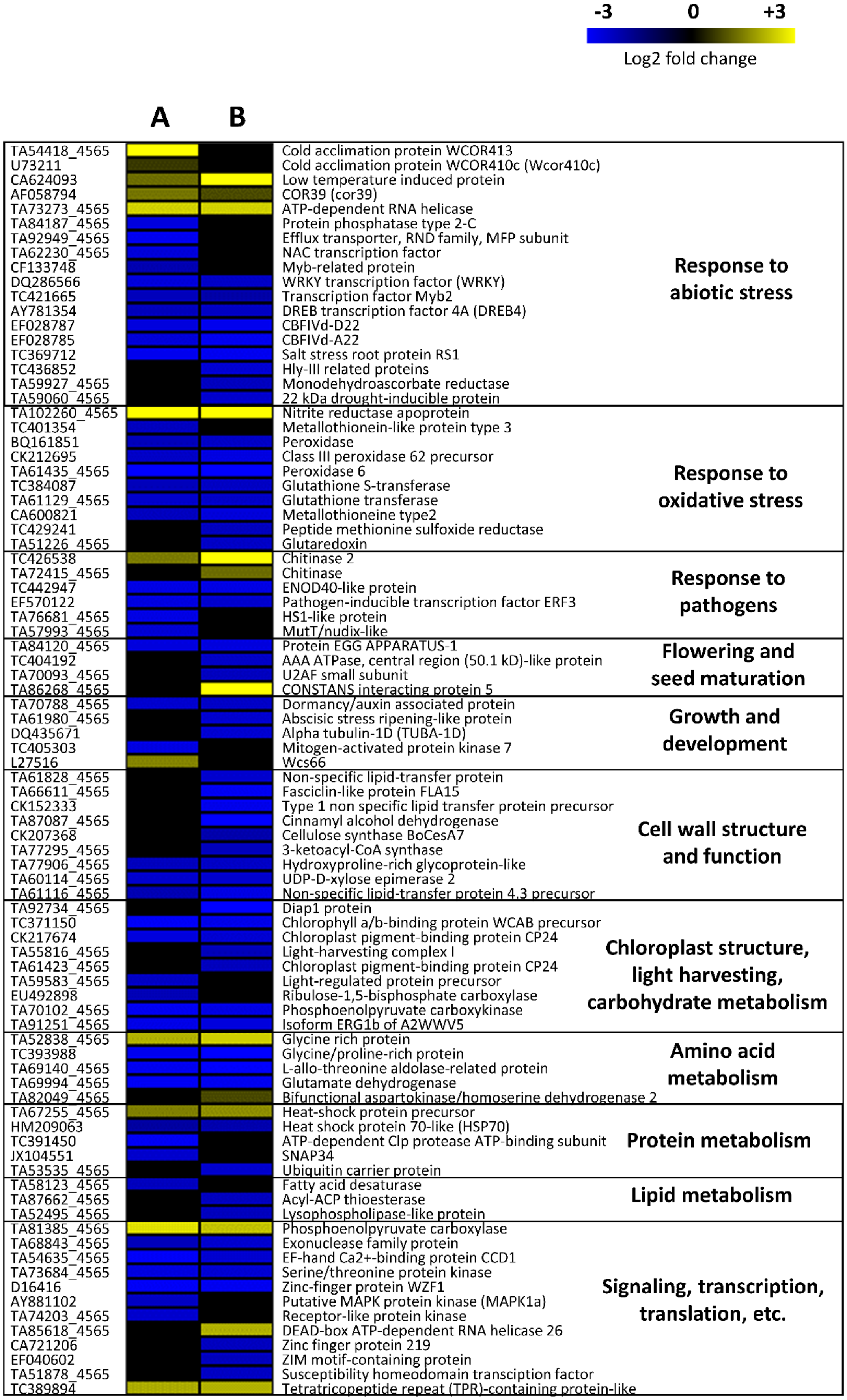
-3
0
+3
Log2 fold change
A
B
TA54418_4565 Cold acclimation protein WCOR413
U73211 Cold acclimation protein WCOR410c (Wcor410c)
CA624093 Low temperature induced protein
AF058794 COR39 (cor39)
TA73273_4565 ATP-dependent RNA helicase
TA84187_4565 Protein phosphatase type 2-C
TA92949_4565 Efflux transporter, RND family, MFP subunit
TA62230_4565 NAC transcription factor
CF133748 Myb-related protein
DQ286566 WRKY transcription factor (WRKY)
TC421665 Transcription factor Myb2
AY781354 DREB transcription factor 4A (DREB4)
EF028787 CBFIVd-D22
EF028785 CBFIVd-A22
TC369712 Salt stress root protein RS1
TC436852 Hly-III related proteins
TA59927_4565 Monodehydroascorbate reductase
TA59060_4565 22 kDa drought-inducible protein
Response to abiotic stress
TA102260_4565 Nitrite reductase apoprotein
TC401354 Metallothionein-like protein type 3
BQ161851 Peroxidase
CK212695 Class III peroxidase 62 precursor
TA61435_4565 Peroxidase 6
TC384087 Glutathione S-transferase
TA61129_4565 Glutathione transferase
CA600821 Metallothioneine type2
TC429241 Peptide methionine sulfoxide reductase
TA51226_4565 Glutaredoxin
Response to oxidative stress
TC426538 Chitinase 2
TA72415_4565 Chitinase
TC442947 ENOD40-like protein
EF570122 Pathogen-inducible transcription factor ERF3
TA76681_4565 HS1-like protein
TA57993_4565 MutT/nudix-like
Response to pathogens
TA84120_4565 Protein EGG APPARATUS-1
TC404192 AAA ATPase, central region (50.1 kD)-like protein
TA70093_4565 U2AF small subunit
TA86268_4565 CONSTANS interacting protein 5
Flowering and seed maturation
TA70788_4565 Dormancy/auxin associated protein
TA61980_4565 Abscisic stress ripening-like protein
DQ435671 Alpha tubulin-1D (TUBA-1D)
TC405303 Mitogen-activated protein kinase 7
L27516 Wcs66
Growth and development
TA61828_4565 Non-specific lipid-transfer protein
TA66611_4565 Fasciclin-like protein FLA15
CK152333 Type 1 non specific lipid transfer protein precursor
TA87087_4565 Cinnamyl alcohol dehydrogenase
CK207368 Cellulose synthase BoCesA7
TA77295_4565 3-ketoacyl-CoA synthase
TA77906_4565 Hydroxyproline-rich glycoprotein-like
TA60114_4565 UDP-D-xylose epimerase 2
TA61116_4565 Non-specific lipid-transfer protein 4.3 precursor
Cell wall structure and function
TA92734_4565 Diap1 protein
TC371150 Chlorophyll a/b-binding protein WCAB precursor
CK217674 Chloroplast pigment-binding protein CP24
TA55816_4565 Light-harvesting complex I
TA61423_4565 Chloroplast pigment-binding protein CP24
TA59583_4565 Light-regulated protein precursor
EU492898 Ribulose-1,5-bisphosphate carboxylase
TA70102_4565 Phosphoenolpyruvate carboxykinase
TA91251_4565 Isoform ERG1b of A2WWV5
Chloroplast structure, light harvesting, carbohydrate metabolism
TA52838_4565 Glycine rich protein
TC393988 Glycine/proline-rich protein
TA69140_4565 L-allo-threonine aldolase-related protein
TA69994_4565 Glutamate dehydrogenase
TA82049_4565 Bifunctional aspartokinase/homoserine dehydrogenase 2
Amino acid metabolism
TA67255_4565 Heat-shock protein precursor
HM209063 Heat shock protein 70-like (HSP70)
TC391450 ATP-dependent Clp protease ATP-binding subunit
JX104551 SNAP34
TA53535_4565 Ubiquitin carrier protein
Protein metabolism
TA58123_4565 Fatty acid desaturase
TA87662_4565 Acyl-ACP thioesterase
TA52495_4565 Lysophospholipase-like protein
Lipid metabolism
TA81385_4565 Phosphoenolpyruvate carboxylase
TA68843_4565 Exonuclease family protein
TA54635_4565 EF-hand Ca2+-binding protein CCD1
TA73684_4565 Serine/threonine protein kinase
D16416 Zinc-finger protein WZF1
AY881102 Putative MAPK protein kinase (MAPK1a)
TA74203_4565 Receptor-like protein kinase
TA85618_4565 DEAD-box ATP-dependent RNA helicase 26
CA721206 Zinc finger protein 219
EF040602 ZIM motif-containing protein
TA51878_4565 Susceptibility homeodomain transciption factor
TC389894 Tetratricopeptide repeat (TPR)-containing protein-like
Signaling, transcription, translation, etc.

SUPPLEMENTARY FIGURE 2 | Heatmap representation of differentially expressed genes (DEGs) in (A) wheat leaves treated with Rh-Est-C12 and infected with *Z. tritici* compared to non-infected and treated leaves (R-D5-i *vs* R-D5-Ni), and (B) the whole response specter of DEGs in wheat leaves treated with Rh-Est-C12 and infected with *Z. tritici* compared to the non-treated and non-infected control plants (R-D5-i *vs* C-D5-Ni), at five days after treatment. Wheat cultivar Alixan, *Z. tritici* T02596 strain and a rhamnolipid concentration of 500 $mg.L^{-1}$ were used. Gene-related physiological processes are represented on the right part of the heatmap and were determined using NCBI, AmiGO 2 Gene Ontology, KEGG and UniProt. Significant relative change in gene transcription is expressed in Log2 ratio, according to the yellow-blue color scale, using the WebMev software. Abbreviations : C stands for a mock treatment while R is for Rh-Est-C12 application ; Ni relates to a mock inoculated wheat while i means inoculated plants with *Z. tritici* ; D5 indicates the sampling date of the third-leaves, corresponding to five days after treatment.

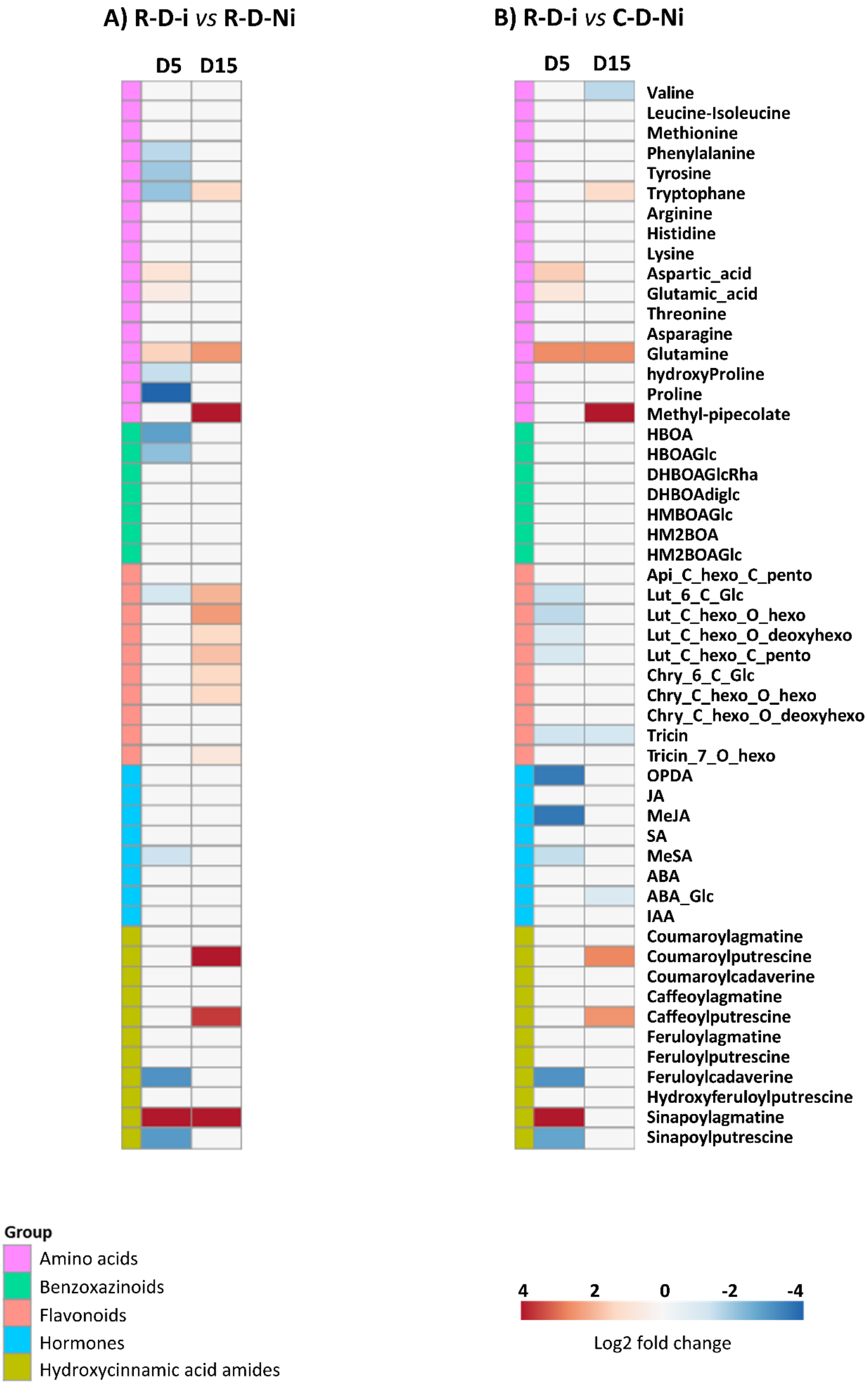
A) R-D-i vs R-D-Ni
B) R-D-i vs C-D-Ni
D5
D15
Valine
Leucine-Isoleucine
Methionine
Phenylalanine
Tyrosine
Tryptophane
Arginine
Histidine
Lysine
Aspartic_acid
Glutamic_acid
Threonine
Asparagine
Glutamine
hydroxyProline
Proline
Methyl-pipecolate
HBOA
HBOAGlc
DHBOAGlcRha
DHBOAdiglc
HMBOAGlc
HM2BOA
HM2BOAGlc
Api_C_hexo_C_pento
Lut_6_C_Glc
Lut_C_hexo_O_hexo
Lut_C_hexo_O_deoxyhexo
Lut_C_hexo_C_pento
Chry_6_C_Glc
Chry_C_hexo_O_hexo
Chry_C_hexo_O_deoxyhexo
Tricin
Tricin_7_O_hexo
OPDA
JA
MeJA
SA
MeSA
ABA
ABA_Glc
IAA
Coumaroylagmatine
Coumaroylputrescine
Coumaroylcadaverine
Caffeoylagmatine
Caffeoylputrescine
Feruloylagmatine
Feruloylputrescine
Feruloylcadaverine
Hydroxyferuloylputrescine
Sinapoylagmatine
Sinapoylputrescine
Group
Amino acids
Benzoxazinoids
Flavonoids
Hormones
Hydroxycinnamic acid amides
4
2
0
-2
-4
Log2 fold change

SUPPLEMENTARY FIGURE 3 | Heatmap representation of differentially expressed genes (DEGs) in (A) wheat leaves treated with Rh-Est-C12 and infected with *Z. tritici* compared to non-infected and treated leaves (R-D5-i *vs* R-D5-Ni), and (B) the whole response specter of DEGs in wheat leaves treated with Rh-Est-C12 and infected with *Z. tritici* compared to the non-treated and non-infected control plants (R-D5-i *vs* C-D5-Ni), at five days after treatment. Wheat cultivar Alixan, *Z. tritici* T02596 strain and a rhamnolipid concentration of 500 $mg.L^{-1}$ were used. Gene-related physiological processes are represented on the right part of the heatmap and were determined using NCBI, AmiGO 2 Gene Ontology, KEGG and UniProt. Significant relative change in gene transcription is expressed in Log2 ratio, according to the yellow-blue color scale, using the WebMev software. Abbreviations : C stands for a mock treatment while R is for Rh-Est-C12 application ; Ni relates to a mock inoculated wheat while i means inoculated plants with *Z. tritici* ; D5 indicates the sampling date of the third-leaves, corresponding to five days after treatment.
